# The Effect of Different pH Conditions on Peptides’ Separation from the Skipjack Dark Meat Hydrolysate Using Ceramic Ultrafiltration

**DOI:** 10.3390/foods12183367

**Published:** 2023-09-08

**Authors:** Supitchaya Pinrattananon, Franck Courtes, Nattawan Chorhirankul, Panwajee Payongsri, Thunyarat Pongtharangkul, Anja E. M. Janssen, Nuttawee Niamsiri

**Affiliations:** 1 School of Bioinnovation and Bio-Based Product Intelligence, Faculty of Science, Mahidol University, Rama 6 Road, Bangkok 10400, Thailand; 2 Global Innovation Center (GIC), Thai Union Group PCL., S.M. Tower, Phaholyothin Road, Phayathai Sub-District, Phayathai, Bangkok 10400, Thailand; 3 Food Process Engineering Group, Wageningen University, P.O. Box 17, 6700 AA Wageningen, The Netherlands; 4 Department of Biotechnology, Faculty of Science, Mahidol University, Rama 6 Road, Bangkok 10400, Thailand

**Keywords:** ultrafiltration, skipjack tuna, pH, transmembrane pressure, peptide transmission

## Abstract

The conversion of Skipjack (*Katsuwonus pelamis*) dark meat into a hydrolysate via enzymatic hydrolysis is a promising approach to increase the value of tuna by-products as a source of bioactive peptides. Skipjack dark meat hydrolysate (SDMH) contains various sizes and sequences of peptides. To obtain and concentrate the targeted small peptides from SDMH, ultrafiltration, a key unit operation process, was employed to fractionate the protein hydrolysate due to its simplicity and productivity. The objective of this study was to investigate the effect of the feed pH on the membrane performance based on the permeate flux and the transmission of peptides. The fractionation of SDMH was performed using a ceramic membrane (molecular weight cut-off of 1 kDa) with three different pH values (5, 7, and 9) at various transmembrane pressures (TMP) (2.85, 3.85, and 4.85 bar). A high permeate flux and transmission were obtained at pH 9 due to the repulsive interactions between peptides and the membrane surface, leading to the reduction in concentration polarization that could promote high transmission. In addition, the combination of low TMP (2.85 bar) and pH 9 helped to even minimize the fouling formation tendency, providing the highest peptide transmission in this study. The fractionation process resulted in the enhancement of small peptides (MW < 0.3 kDa). The amino acid profiles were different at each pH, affirming the charge effect from the pH changes. In conclusion, the performance of the membrane was affected by the pH of the hydrolysate. Additionally, the ultrafiltration method served as an alternate method of peptide separation on a commercial scale.

## 1. Introduction

The world’s aging population has made us more aware of living with a good and healthy quality of life [1]. Growing older is usually accompanied by deteriorating health and leads to different diseases. Those health-related issues are personal, which results in variations in individual treatment. Hence, functional foods and nutraceuticals have received much attention, especially for their health benefits to help protect against certain chronic diseases, especially cancer, cardiovascular diseases, gastrointestinal tract disorders, and neurological diseases. Bioactive peptides are interesting functional food alternatives for those purposes due to their biological properties, including anti-inflammation, anti-hypertension, anti-obesity, and anti-oxidation [2]. In addition, the bioactive peptides also show low side effects compared to conventional chemical drugs since they are from various food protein sources. Typically, bioactive peptides are specific small protein fragments generated via hydrolysis and usually contain 2–20 amino acids with a frequent presence of hydrophobic amino acids. Moreover, several studies have claimed that the peptides containing proline, lysine, and arginine groups exhibit bioactivities [3,4,5]. The bioactive peptides could be produced from dairy-, plant-, and animal-protein sources that can provide different bioactivities depending on the size and sequence of peptides [2].

Tuna (*Thunnus* spp.) and tuna-like species have long been recognized as major fishery commodities. Skipjack tuna (*Katsuwonus pelamis*) is about 58% of the total caught fish for tuna processing, which is annually more than 2 million metric tons. During the canning process, almost 70% of the parts of a fish are considered by-products including blood, head, viscera, bone, skin, and dark meat [6]. The dark meat is nutritious and rich in high-quality proteins. However, due to the higher lipid content, heme protein concentration, and lower pH values, the rapid deterioration and oxidation can often occur, leading to a darker color and fishy smell making the meat less attractive for use in fresh or canned products [7,8]. On the other hand, the dark meat can be effectively transformed via enzymatic hydrolysis, converting dark meat proteins into many value-added products, including the generation of functional bioactive peptides.

Skipjack dark meat hydrolysate (SDMH) consists of various peptides, but not all peptides have bioactivity. The bioactivities of peptides depend on their size, sequence, and conformation [9,10,11,12,13]. Studies reported that peptides from tuna dark meat hydrolysate with a molecular weight of less than 1 kDa (i.e., around 10 amino acids or less) showed great bioactivities such as ACE inhibition, anti-oxidation, and anti-cancer [10,11,12,14]. Hence, in the production of bioactive peptides from SDMH, a unit operation involving the size separation is necessarily employed to fractionate small-size peptides.

To separate a complex mixture of different-size peptides based on the size or molecular weight separation, many technologies have been employed, such as liquid chromatography, gel filtration chromatography, ion exchange chromatography [15], and aqueous two-phase extraction [16,17], which provide high selectivity [18,19,20]. However, most of these separation technologies have high operating costs for large-scale production [21]. As an alternative option for peptide separation, ultrafiltration, which is a membrane separation process based on size, has a great potential. This process provides mild operating conditions, a simple scaling-up process, high productivity, and a lower operating cost [22]. Membrane technology has also been effectively used for food applications to enrich specific sizes of molecules from various by-product hydrolysates.

Several studies reported that the membrane process performance was affected by operation conditions, which are transmembrane pressure (TMP), temperature and solution properties such as pH, ionic strength, concentration, etc. [18,23,24,25,26,27,28,29]. An ultrafiltration is not only based on the size of the molecules, but also the charges of the peptides and the membrane affect the selectivity. According to Fernández et al. and Saidi et al., the charge interactions between peptides and membranes could be affected by the influence of the solution pH that subsequently influenced both the solute transmission and the permeate flux [24,30]. Moreover, charge interactions from a pH change have an impact on concentration polarization and fouling, which subsequently affect the permeability and fractionation of peptides. In addition, membrane material also impacts the separation performance [26]. Among the materials, ceramic provides a long life span, high stability (pH, chemical, thermal, and mechanical), a low operation and maintenance cost, and easy and fast cleaning [31]. With these advantages, ceramic is the interesting option for separation on the industrial scale. However, only few studies have investigated the effect of operation conditions on the SDMH separation, especially charge interaction regarding the feed pH modification using a ceramic membrane. In addition, studying a larger scale ultrafiltration process is important to understand for process development and operation at the pilot and industrial levels [25,30,32,33].

This study aimed to investigate the effect of pH (5, 7, and 9) at various TMP (2.85, 3.85, and 4.85 bar) on the SDMH separation performance of an ultrafiltration process. The experiment was performed by using a tubular ceramic membrane with a molecular weight cut-off (MWCO) of 1 kDa. The permeate flux and transmission were determined to show the membrane performance under the influence of pH and in combination with TMP. The protein concentration, molecular weight distribution (MWDB), and amino acid profiles were measured in retentates and permeates.

## 2. Materials and Methods

### 2.1. Materials

Thyroglobulin (664,000 Da), Cytochrome C (12,327 Da), Aprotinin (6511 Da), Bacitracin (1423 Da), N-Hippuryl-His-Leu hydrate (HHL) (429 Da), Glycine-Glycine (132 Da), Folin and Ciocalteu’s phenol reagent, and Trifluoroacetic acid (TFA) were purchased from Sigma Chemical Co. (St. Louis, MO, USA). HPLC-grade acetonitrile was purchased from Fisher Scientific (Nepean, ON, Canada). All chemicals used in this study were analytical grade.

### 2.2. Preparation of Protein Hydrolysate Feed

SDMH was produced and clarified by the Thai Union Group PCL, Thailand. After receiving, the hydrolysate was aliquoted (7 L per container). Then, the hydrolysate was kept at −20 °C until use. The proximate compositions and peptide fractions of SDMH are reported in Table 1.

### 2.3. Ultrafiltration Experiment

#### 2.3.1. pH Modification and Pre-Filtration

The frozen hydrolysate was thawed, and the pH of the solution was adjusted to the desired value of 5, 7 (control), and 9 using 6 M of HCl and/or 6 M of NaOH. Then, the adjusted solution was filtered using the Whatman^®^ glass microfilter grade GF/D (Whatman plc, Buckinghamshire, United Kingdom) to remove large particles and dust.

#### 2.3.2. Membrane

A tubular ceramic membrane with a MWCO of 1 kDa was purchased from Atech, Germany. The filter surface area of the membrane is 0.00475 m^2^ with a point of zero charge of pH 4.6 [38]. The membrane materials are ZrO_2_ and Al_2_O_3_ and the support material is α-Al_2_O_3_.

#### 2.3.3. Hydrolysate Filtration Experiment

**Figure 1:**
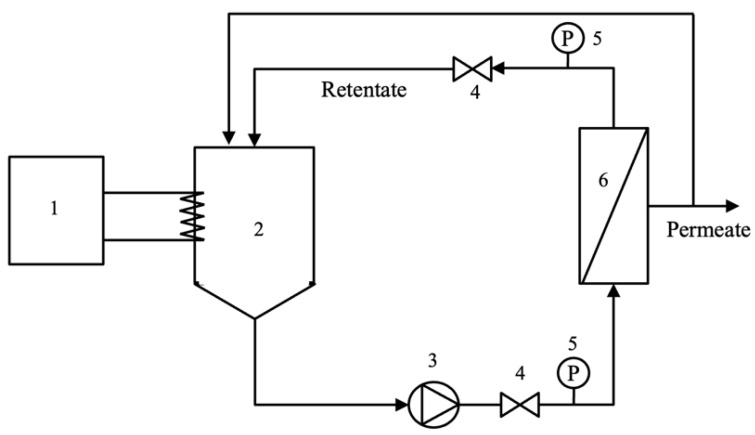
Ultrafiltration experiment set-up (1: thermostat; 2: feed tank; 3: pump; 4: valve; 5: pressure gauge; and 6: membrane module).

The pre-filtrated hydrolysate (7 L) was fed into the ultrafiltration (UF) system as shown in Figure 1. The retentate flow rate and pressure drop across the membrane module were controlled via valve number 4 (Figure 1). The hydrolysate filtration experiment was carried out in a tangential filtration mode using the ceramic membrane. The permeate and retentate streams were recycled back to the feed tank at a controlled temperature of 50 °C. Retentate flow rate and pressure drop across the membrane were fixed at 7.5 L/min and 0.3 bar, respectively. Each TMP condition (2.85, 3.85, and 4.85 bar) was operated for a total running time of 90 min. After 50 min, the permeate and retentate were collected for further analysis. The permeate flux was determined at a steady state by measuring the permeate flow rate (for 9 min) at 50, 60, 70, 80, and 90 min of operation. All experiments were performed in three replicates.

#### 2.3.4. Membrane Cleaning

The membrane was cleaned after each run by following the manufacturer’s recommendation (Atech, Germany). The cleaning steps included rinsing the membrane with water (under a pressure of 2 bar) until the pH became neutral, and a basic wash (1% NaOH) at a pressure of 3 bar and a temperature of 80 °C for 60 min. Then, the membrane was rinsed with water (under a pressure of 2 bar) until the pH became neutral. After that, the acid wash (0.3% HNO_3_) was performed at a pressure of 3 bar and a temperature of 30 °C for 15 min. Lastly, the membrane was rinsed with water (at a pressure of 2 bar) until the pH became neutral. The temperature during each cleaning step was controlled to prevent thermal shock that could lead to membrane damage. The maximum temperature difference should be lower than 30 °C and the temperature change rate should be less than 10 °C per minute. Next, the membrane was stored in deionized water prior to the next run. The water permeability of the membrane was measured every time before performing a filtration experiment. The pressure drop, flow rate, and temperature were fixed at 0.3 bar, 7.5 L/h, and 40 °C, respectively. According to [39], the slope of the water flux as a function of TMP was the water permeability value. The water permeability of the cleaned membrane was controlled at ±20% of the cleaned unused membrane (49.23 L/m^2^h bar). After completing all experiments, the membrane was always cleaned and stored in a dry condition.

### 2.4. Analysis

#### 2.4.1. Proximate Compositions and Protein Concentration

Proximate compositions of the SDMH (original peptides’ mixture) were determined according to the Association of Official Analytical Chemists [34]. Crude protein was measured according to Kjeldahl’s method. The total nitrogen content was calculated with 6.25 as the conversion factor. The moisture content of SDMH was determined by drying in a hot air oven. The fat content was determined via a Soxhlet method [10,35]. Ash content was estimated by burning in a furnace [36]. The protein concentration of the SDMH fraction from the ultrafiltration experiment was determined according to the Lowry method with bovine serum albumin (BSA) as a standard [35,36,40].

#### 2.4.2. Molecular Weight Distribution

The MWDB of the hydrolysate samples was determined with high-performance liquid chromatography (HPLC). A gel filtration column, TSKgel^®^G2000SWXL 7.8 × 300 mm column (Tosoh, Tokyo, Japan), was used. The system was a 1260 Infinity system equipped with a diode-array detector (Agilent, CA, USA) according to Gu’s method [37]. The calibration curve was constructed using Cytochrome C (12,327 Da), Aprotinin (6512 Da), Bacitracin (1423 Da), N-Hippuryl-His-Leu (429 Da), and Gly-Gly (132 Da). Forty-five percent of Acetonitrile with 0.1% trifluoroacetic acid (TFA) was used as a mobile phase at a constant flow rate of 0.5 mL/min. The filtered samples were injected with a 20 µL volume and eluted at 30 °C. The diode-array detector detected absorbances at 220 nm [37]. The area under the chromatogram (*AC*) was integrated and separated into three molecular weight (MW) ranges which are <0.3, 0.3–1, and >1 kDa, respectively. The total peptide transmission of peptides (*Tr*) was calculated for every pH and transmembrane pressure condition using Equation (1).

(1) $$Tr=\frac{{AC}_{p}}{{AC}_{r}}\times 100$$

where *AC_p_* and *AC_r_* are the areas under the chromatogram of permeate and retentate, respectively [24].

#### 2.4.3. Amino Acid Profiles

The amino acid profiles were performed by a certified laboratory ALS group (Thailand) according to the AOAC method. To generate the amino acids, an acid hydrolysis was employed using 6 M of HCl (sample to acid solution at a ratio of 1:100) at 100 °C for 24 h. After that, the amino acids were analyzed via HPLC [34].

#### 2.4.4. Statistical Analysis

The statistical analysis was performed using SPSS software (IBM SPSS Statistics 29, 2022, International Business Machines (IBM), Armonk, NY, USA)). The results were subjected to the analysis of variance (ANOVA) combined with an LSD post-hoc test, and the means were compared at a significant level of 0.05.

## 3. Results and Discussion

### 3.1. Effects of pH Adjustment and Pre-Filtration on Hydrolysate Characterization

Before the ultrafiltration experiments, the SDMH was adjusted to the desired pH and filtered using filter paper. Thus, the SDMH (original peptides’ mixture) and the feeds (SDMH after pH modification and prefiltration) were characterized to clarify the effect of pH modification and pre-filtration.

**Figure 2:**
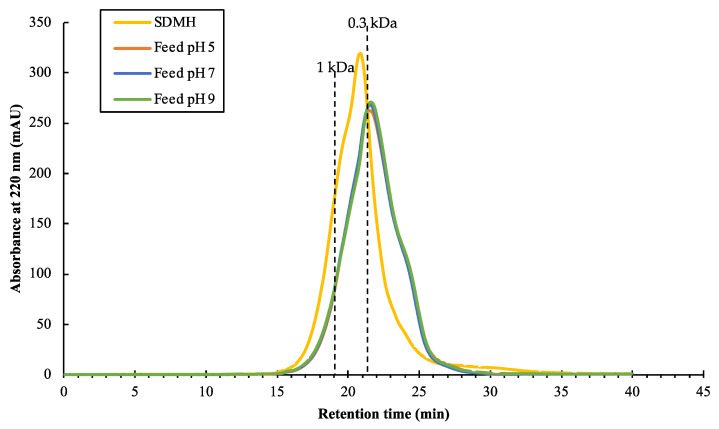
Chromatograms of SDMH and the feeds at different pH values.

The characterizations of the SDMH and the feeds were investigated via the protein concentration, MWDB, and the amino acid profiles. After pre-filtration, a fouling layer was observed on the filter paper. At pH 5, the filter paper showed less fouling as compared to the filters at pH 7 and 9 (See Supplementary Data Figure S1). Table 2 reports the protein concentration between SDMH and the feed solutions. The protein concentration of the feeds at different pH values showed no significant difference (*p*-value > 0.05). This finding suggested that the pH modification and pre-filtration had no significant impact on the change in protein concentration. However, the MWDB of SDMH was different from the feeds at all pH values as shown in Figure 2. The peptides with MW > 0.3 kDa were more present in the original peptides’ mixture (SDMH) than the feeds. Thus, the pH modification and pre-filtration step could affect the size of peptides in the hydrolysate solution by removing some of the MW > 0.3 kDa peptides. Figure 2 demonstrated that the feeds at three pH values had a relatively comparable MWDB of peptides, with roughly 58%, 35%, and 7% of peptides with a molecular weight range of less than 0.3 kDa, 0.3–1 kDa, and higher than 1 kDa, respectively. This finding suggested that the different pH values of the feed caused no differences of the MWDB of peptides.

In addition, the amino acid profiles were determined to exhibit the effects of pH modification and pre-filtration. The amino acid profiles are shown in Table 3. The compositions revealed that the feed solutions at pH 7 and pH 9 had similar trends as SDMH while the feed at pH 5 exhibited different percentage compositions. The amount of amino acids with an aromatic side chain were reduced from 6.2% to 1.6% while proline, a unique amino acid, was decreased from 23.1% to 6.4% after pH modification to pH 5 and pre-filtration. It was possible that the peptides containing amino acids with an aromatic side chain or proline might aggregate at pH 5 as the isoelectric point of both amino acids with an aromatic side chain and proline are in the range of 5.4–6.3. However, both amino acids with electrically charged acidic- and basic-side chains of the feed at pH 5 were increased from 16.3% to 22.2% and from 17.9% to 29.6%, respectively.

In addition to the amino acid compositions of SDMH and the feeds, there were different fouling thicknesses found on the used filtration paper at different pH values, and pH 5 showed the least fouling thickness on the filter paper (See Supplementary Data Figure S1). These observations support that the charges of peptides might be changed after pH adjustment and has an impact on the aggregation of protein and peptides as evidence on the filter paper [41]. At pH 5, the higher transmission of solutes (i.e., amino acids, peptides, and proteins) was obtained since the pH 5 hydrolysate had less aggregation than pH 7 and 9. This could indicate that the high MW peptides at pH 5 might gain more charges and had a high electrostatic interaction which resulted in lower aggregation.

Thus, using the pH modification and prefiltration as the hydrolysate preparation steps before running the ultrafiltration affect the amino acid compositions in the feeds and the size distribution of peptides. However, the protein concentrations of hydrolysate before and after the pH modification and pre-filtration steps showed no changes.

### 3.2. Effects of Feed pH and Transmembrane Pressure on Permeate Flux

**Figure 3:**
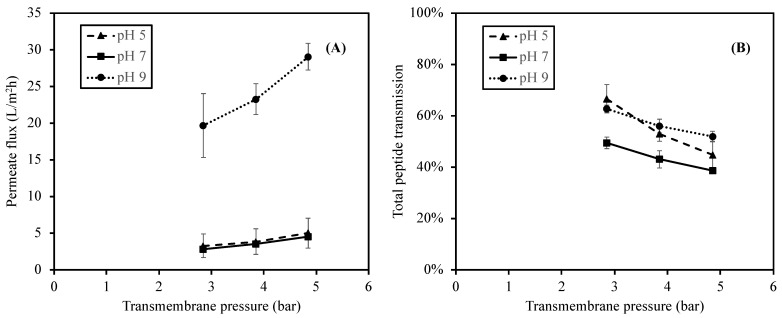
Effect of feed pH on permeate fluxes (**A**) and total peptide transmission (**B**) when using 1 kDa ceramic membrane at the TMP of 2.85–4.85 bar.

The influences of the feed pH and TMP on permeate flux were studied under three pH values of 5, 7 (control), and 9, representing acid, neutral, and alkaline conditions at three different TMP values (2.85, 3.85, and 4.85 bar). The effect of pH on the permeate fluxes at different TMP values is summarized in Figure 3.

As shown in Figure 3, the lowest permeate fluxes were obtained at pH 5 and 7 with values ranging from 3.3 to 5.0 L/m^2^h and from 2.0 to 4.5 L/m^2^h, respectively. The permeate fluxes increased significantly (*p*-value < 0.05) as the pH increased to pH 9 with the values from 19.7 to 29.1 L/m^2^h at the same range of TMP. The result suggested that the change in the feed pH to pH 9 (alkaline) significantly increased the permeate flux. This observation was in accordance with the studies reported by [30] on the Prolastin^®^ hydrolysate solution and by [33] on tilapia by-product hydrolysates. Both studies reported high electrostatic repulsive interactions of peptide–peptide and peptide–membrane due to the negative charges of peptides and the membrane surface (point of zero charge of 4.6) in alkaline pH conditions [38]. As a result, the high electrostatic interactions reduced the tendency of protein aggregation on the membrane surface, and therefore promotes high permeability of the hydrolysate solution.

Normally, there is high protein aggregation and protein adsorption on the membrane surface when the electrostatic interactions between peptide–peptide and peptide–membrane were low. At pH 5 and 7, the lower permeate fluxes suggested the lower repulsive interactions from the negative peptides and the membrane surface. As the pH was reduced, some peptide charges became more positive, resulting in no net charge or even a positive charge from those peptides. Thus, the charge interactions (repulsive interactions) were less, and they were assumed that the hydrophobic interactions between zero net charge peptides increased. This might lead to an increase in membrane fouling as well as a reduction in the permeate flux [33,42].

In addition to the pH, as the membrane filtration was a pressure-driven process, the TMP also affected the permeate flux (Figure 3). The permeate flux increased continuously with an increase in TMP. The permeate fluxes at a TMP of 4.85 bar were remarkably higher (*p*-value < 0.05) than those at a TMP of 2.85 and 3.85 bar (which were not significantly different; *p*-value > 0.05). This phenomenon could be observed at every pH value. As TMP is a pressure difference between the feed and permeate stream which forces the fluid through the membrane [43], an increase in the permeate flux was generally observed at a higher TMP. The results obtained in this study agreed with the reports from [30,42,44], in which high TMP could promote high permeate fluxes of the hydrolysate solutions.

In summary, the obtained results clearly revealed that the alkaline pH significantly increased the permeate flux of hydrolysate, possibly as a result of the strong electrostatic interactions of negative charge peptides and the membrane surface, which resulted in the reduction in protein aggregation. Lower pH conditions, on the other hand, seemed to have less repulsive interactions and a higher tendency of fouling layer formation.

### 3.3. Effect of pH and Transmembrane on Protein Concentration and Transmission

In this study, a filtration of SDMH was performed using different combinations of pH and TMP. The permeability of peptides to the permeate side can be observed from the protein concentrations in the feed and permeate (Table 4). As the protein concentration of the feed at different pH values are not significantly different (*p*-value > 0.05), it can be concluded that the pH modification performed in this study did not affect the feed protein concentration. The permeate from each combination showed approximately 50–60% lower protein concentration than the corresponding feed. At a TMP of 2.85 bar, the protein concentration of permeate from different pHs showed no significant difference, whereas at a TMP of 3.85 and 4.85 bar, the permeate from pH 5 and 9 showed a significantly higher protein concentration than pH 7. Furthermore, a significant reduction in protein concentration in the permeate was observed at all pH values when the TMP was increased from 2.85 to 3.85 and 4.85 bar (*p*-value < 0.05).

In addition to the protein concentration, the effect of pH was similarly observed in the total peptide transmission result. As shown in Figure 3, the total peptide transmission at pH 5 and 9 are not significantly different (*p*-value > 0.05), and the total peptide transmission at pH 7 is significantly lower (*p*-value < 0.05) than those at pH 5 and 9. Moreover, the total peptide transmission of peptides significantly decreased when the TMP increased from 2.85 to 4.85 bar (*p*-value < 0.05), regardless of pH. 

Regarding the effect of pH, both protein concentrations in permeates and the total peptide transmissions of peptides, which all indicted the performance of the membrane, was higher at pH 5 and 9. Several studies reported that the transmission was influenced by the charge interactions, electrostatic double layer, and conformation of protein, which highly depends on pH [27,30,33,41,44]. The high transmission of peptides was usually obtained when the electrostatic interactions between peptide–peptide and peptide–membrane surface were high. For the alkaline pH, the peptides obtained higher negative charges. Thus, the higher repulsive interactions between negatively charged peptides and the membrane surface reduced the tendency of protein accumulation on the membrane surface. For a pH of 5, a higher peptide transmission was expected as a result of the high electrostatic interactions between positively charged peptides and the positively charged dynamic membrane layer, which resulted in less protein aggregation and accumulation [45]. It should be noted that the permeate flux at pH 5 was lower than that at pH 9 (Figure 3), which implied less solution (i.e., water and peptides) transmission at pH 5. As pH highly affected the peptide charges and their attractive/repulsive interactions, a different transmission was therefore expected.

In addition to the pH, TMP also influenced the permeability of hydrolysate as it was the pressure that pushed the solute across the membrane. Unlike the permeate flux which increased at a higher TMP (Figure 3), the protein concentration in permeates and the total peptide transmission decreased at a higher TMP, indicating that the higher pressure applying to the membrane affected the concentration polarization and fouling on the membrane surface. Hence, the solutes, which were peptides, tended to accumulate at the membrane surface. Therefore, the concentration polarization limited the transmission of solutes [25,46,47]

### 3.4. Effect of pH and Transmembrane Pressure on Size Separation

**Figure 4:**
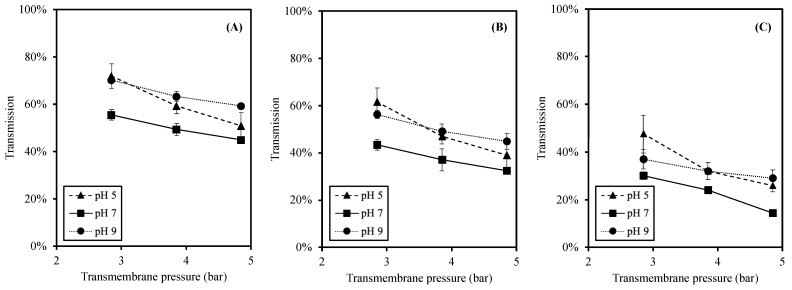
Effects of pH and TMP on transmission of each MW peptide where (**A**) MW < 0.3 kDa; (**B**) MW 0.3–1 kDa; and (**C**) MW > 1 kDa.

To investigate the size separation of this membrane process, the transmissions of peptides are reported in three different molecular weight (MW) size ranges of less than 0.3 kDa (<0.3 kDa), between 0.3 and 1 kDa (0.3–1 kDa), and higher than 1 kDa (>1 kDa), as shown in Figure 4. The peptides with MW < 0.3 kDa exhibit the highest transmission among the size ranges. The significant reduction in transmission was observed when the size of peptides were bigger (*p*-value *<* 0.05). These trends were noticed at all pH values.

In accordance with the total peptide transmission (Figure 3), the effects of TMP and pH on the transmission of specific size peptides (Figure 4) were in similar trends. First, the transmission was reduced at higher TMP as a result of a higher peptide accumulation and fouling at the membrane surface [25,46]. Second, a significantly higher transmission was observed at pH 5 and 9 (than pH 7) at all sizes of peptides. Due to the pH changes, the charges of peptides changed accordingly, leading to different electrostatic interactions [27,30,33,41,44]. The higher electrostatic interactions resulted in a high transmission and less peptide accumulation at acidic and alkaline pH [30,33,45].

The peptides with MW < 0.3 kDa provided the highest transmission, whereas the peptides with MW 0.3–1 kDa, which were smaller than MWCO, were significantly lower (Figure 4). This result agreed well with the report by [25], in which the MWDB of higher MW peptides was lower at higher TMP. Since the concentration polarization increased at high TMP, the apparent MWCO of the membrane was reduced [48]. Ref. [30] studied the effect of TMP on hydrolysate filtration, and the results showed that increasing the TMP led to a higher transmission of peptides having MW less than the membrane MWCO. The observed phenomenon in which the peptides with MW > 0.3 kDa were less likely to transmit through the membrane used in this study indicated the reduction in apparent MWCO and the high concentration polarization. Thus, the performance of the membrane in this study provided more the separation of small MW peptides (MW < 0.3 kDa).

### 3.5. Amino Acid Profiles of Hydrolysate at Different pH Values

To investigate the effect of pH on the separation by UF, the total amino acid compositions of the hydrolysate samples were analyzed. The amino acid compositions of the feeds, permeates, and retentates at pH 5, 7, and 9 are summarized in Table 5. The amino acid profiles of permeates and retentates were obtained from an operation at TMP of 2.85 bar, which exhibited the highest transmission. After ultrafiltration, it was observed that the amino acid profiles of the permeates and retentates at pH 5, 7, and 9 were similar. However, there were slight differences observed in the permeates which included slightly higher levels of alanine in permeate pH 9, and glutamic acid in permeate pH 7. Moreover, no cysteine was found in the permeates at all pH values.

As cysteine was already low in the feed, none of cysteine was observed in permeates at all pH values which indicated that cysteine was exclusively retained in the retentate. There was a chance that peptides containing cysteine had strong charge interactions with other peptides and membrane surface or cysteine might be present in the high MW peptides (i.e., higher than MWCO) which was unable to transmit through the membrane and therefore remained in the retentate. On the other hand, there was a potential that peptides containing alanine and glutamic acid were present in small peptides or had higher attractive charge interactions which resulted in slightly higher percentage of amino acids in permeate. Thus, this indicated the change in peptide compositions in the hydrolysate during ultrafiltration process.

Permeate-to-retentate (P/R) ratio is used as indicator of separation performance by UF. P/R can also be considered as the transmission which compared the amino acid concentration of permeate and retentate which are summarized in Table 6. It should be noted that the P/R of many amino acids at different pH values showed similar trends. Nevertheless, some amino acids slightly differed comparing between pH values such as acidic charged side chains (aspartic acids and glutamic acids), and basic electrical charged side chain (histidine) that higher at pH 7, while arginine provided the lowest P/R at pH 5.

According to electrostatic charge interactions, hydrophobic-aliphatic and polar neutral side chain amino acids had isoelectric points slightly higher than pH 5, and electrically charged side chain-acidic amino acids had isoelectric points lower than pH 5. Thus, at pH 7 and 9, the amino acids became more negatively charged. As a result, the negatively charged amino acids, peptides, and the membrane surface contributed to repulsive interactions and a higher transmission [33]. Moreover, the acidic-side-chain amino acids with negative charges (aspartic acids and glutamic acids) had a higher P/R at pH 7 than pH 9. This could imply that the electrostatic interactions between peptides containing aspartic acids and glutamic acids were higher, resulting in more transmissions (P/R) of the amino acids.

In addition, electrically charged side-chain-basic amino acids at pH 7 and 9 showed a less positive charge than pH 5. Thus, attractive interactions between peptides containing these amino acids at pH 7 and 9 were less than pH 5, which showed stronger positive charges (further away from the isoelectric point of these amino acids) [47]. Hence, the higher P/R of arginine was observed at pH 7 and 9. However, the P/R ratios of histidine were similar at pH 5 and pH 9, which were lower than pH 7. With this finding, it was possible that peptides containing histidine had a greater transmission at pH 7, which might be from a higher electrostatic interaction.

These observations of amino acid compositions and P/R suggested that the ceramic membrane filtration of SDMH in this study produced slightly different amino acid profiles of the permeates and retentates at any pH values, and there were different amino acid profiles of the feeds at various pH values. These supported the fact that the pH altered the membrane separation in this study. In addition to the amino acid results, the permeate flux and transmission results also exhibited differently at various pH values. The lowest peptide transmission (Figure 4) and the permeate flux (Figure 3) were observed at pH 7. At this pH, the peptides had less negative and positive charge than at pH 9 and pH 5, respectively. As a result, the repulsive interactions were low, but hydrophobic interactions between non-polar peptide groups would be enhanced, resulting in a higher protein accumulation on the membrane surface. Thus, the transmission of peptides as well as the permeate flux (including solutes and solvent) were decreased [24,44].

The amino acid profiles showed that the feed pH modification and pre-filter using filter paper caused amino acid composition changes. Moreover, each amino acid behavior varied with the feed pH due to charge interactions. On the other hand, these amino acid profiles were analyzed based on the amino acids in both the free amino acid and the amino acid present in peptides. The peptide sequences are still unknown. As a result, the amino acid profiles implied the indirect effect of pH on peptide transmission.

## 4. Conclusions

Fractionation of SDMH was successfully performed using the 1 kDa ceramic UF membrane in tangential mode. The performance based on the permeate flux and the peptide transmission values was influenced by the TMP and pH. The TMP influenced the permeate flux and peptide transmission. Increasing of the TMP increased the permeate flux. Due to concentration polarization and apparent MWCO reduction, however, low peptide transmission was obtained at high TMP. Furthermore, the permeate was enriched in peptides with MW less than 0.3 kDa while retaining the peptides with higher MW. These enriched 0.3 kDa peptides in the permeate could be tested for potential bioactivities including anti-hypertension, anti-oxidant, and anti-cancer properties. In addition to the TMP, the feed pH also affected the fractionation performance resulting from the change in peptide charges and electrostatic interactions. The highest permeate flux was obtained at the alkaline pH, which had high repulsive interactions from the negatively charged peptides and membrane surface, while pH 5 and 7 were lower and did not differ significantly. Furthermore, peptide transmission was significantly higher at pH 5 and 9 than at pH 7. The pH changed from the control pH of 7, which affected the peptide charges and resulted in different charge interactions. Thus, the pH 9 and 5 had higher repulsive interactions from the negatively charged peptides–membrane surface and the positively charged peptides–positively charged peptide layers on the membrane surface, respectively. Hence, the concentration polarization was reduced and led to a higher peptide transmission. While at pH 7, the peptides had less charges than pH 9 and 5, respectively. Then, there were fewer repulsive interactions but higher hydrophobic interactions, which increased the concentration polarization and peptide accumulation tendency. Moreover, the amino acid profiles revealed that this ultrafiltration process produced permeates and retentates that have no significantly different amino acid profiles. However, the feed pH modification and prefiltration caused the change in amino acid profiles at pH 5. On the other hand, the transmission of amino acids at different pH values was little altered, as shown in the permeate and retentate amino acid profiles where some amino acids were different at the altered pH values. Hence, the main conclusion was that pH had an impact on the membrane performance in this study. The conditions providing high permeate flux and high peptide transmission were alkaline pH and low TMP, in which the concentration polarization and fouling effects were minimized. Lastly, the ultrafiltration process was an interesting alternative approach for peptide separation on industrial scale production.

## Figures and Tables

**Table 1:** Proximate compositions and MWDB of SDMH (original peptides’ mixture).

Proximate Compositions	g/100 g	References
Protein	5.6	[34]
Fat	0.2	[10,34,35]
Carbohydrate	0.2	[34]
Ash	0.6	[34,36]
Moisture	93.4	[34]
Peptide fraction	%	[37]
<0.3 kDa	54.5	
0.3–1 kDa	36.8	
>1 kDa	8.7	

**Table 2:** Protein concentration of SDMH and the feeds at different pH values.

	**SDMH**	**Feed**		
		**pH 5**	**pH 7**	**pH 9**
Protein concentration * (g/L)	37.6 ± 0.7 ^a^	39.2 ± 0.2 ^a^	37.3 ± 0.2 ^a^	37.2 ± 2.1 ^a^

Note: * Protein concentrations were determined according to Lowry’s method, ^a^ indicated no significant difference *p*-value > 0.05) in protein concentrations among samples.

**Table 3:** Amino acid compositions of SDMH and the feeds at different pH values.

Amino Acids	SDMH		Feeds					
			pH 5		pH 7		pH 9	
	AV	%	AV	%	AV	%	AV	%
**Hydrophobic ** **(aliphatic)**	1.5	27.0	1.5	27.1	1.3	27.5	1.2	28.9
Valine	0.2	3.9	0.1	2.4	0.2	3.6	0.2	3.7
Isoleucine	0.2	2.9	0.1	2.2	0.1	2.8	0.1	2.9
Leucine	0.6	11.0	0.7	13.1	0.6	11.4	0.5	11.6
Methionine	0.2	3.3	0.2	3.8	0.2	3.8	0.2	4.9
Alanine	0.3	5.9	0.3	5.6	0.3	5.9	0.3	5.8
**Hydrophobic ** **(aromatic)**	0.3	6.2	0.1	1.6	0.3	5.9	0.3	5.9
Phenylalanine	0.2	3.7	0.1	0.8	0.2	3.7	0.2	3.8
Tryptophan	0.0	0.0	0.0	0.0	0.0	0.0	0.0	0.0
Tyrosine	0.1	2.5	0.0	0.8	0.1	2.3	0.1	2.1
**Polar neutral**	0.4	6.4	0.4	7.7	0.3	6.3	0.3	6.4
Threonine	0.2	3.2	0.2	4.2	0.2	3.1	0.1	3.2
Serine	0.1	2.4	0.2	2.6	0.1	2.4	0.1	2.5
Cysteine	0.1	0.9	0.1	0.9	0.0	0.8	0.0	0.7
**Electrical charged (acidic)**	0.9	16.3	1.3	22.2	0.8	15.6	0.6	14.7
Aspartic acid	0.4	7.1	0.8	13.3	0.3	6.7	0.3	6.5
Glutamic acid	0.5	9.2	0.5	9.0	0.4	8.8	0.4	8.2
**Electrical charged (basic)**	1.0	17.9	1.7	29.6	0.9	18.0	0.7	17.6
Histidine	0.2	4.2	0.7	12.3	0.2	4.2	0.2	4.0
Lysine	0.4	7.9	0.7	13.2	0.4	8.6	0.4	8.5
Arginine	0.3	5.9	0.2	4.1	0.3	5.2	0.2	5.1
**Unique**	1.5	26.3	0.7	11.8	1.3	26.7	1.1	26.6
Glycine	0.2	3.2	0.3	5.4	0.2	3.5	0.2	3.8
Proline	1.3	23.1	0.4	6.4	1.1	23.2	1.0	22.8
**Total**	5.5	100.0	5.6	100.0	4.9	100.0	4.2	100.0

Av: Average amino acid concentration (g/100 g sample); %: Percentage composition.

**Table 4:** Protein concentration of Skipjack dark meat hydrolysate (SDMH) feed and permeate.

	Protein Concentration (g/L)			
	Feed	Permeate		
**TMP (bar)**		**2.85**	**3.85**	**4.85**
pH 5	39.2 ± 0.2 ^a^	16.4 ± 0.7 ^a,A^	15.0 ± 1.0 ^a,B^	13.6 ± 0.1 ^b,B^
pH 7	37.3 ± 1.6 ^a^	16.2 ± 0.9 ^a,A^	12.4 ± 0.4 ^b,B^	11.9 ± 0.6 ^c,B^
pH 9	37.2 ± 2.1 ^a^	17.3 ± 0.6 ^a,A^	16.0 ± 0.7 ^a,B^	15.2 ± 0.3 ^a,B^

Note: Different lowercase alphabet superscripts indicate a statistically significant difference (*p*-value < 0.05) in protein concentration between different pH values (at a fixed TMP of 2.85, 3.85, or 4.85 bar) in the same column. Different uppercase alphabet superscripts indicate a statistically significant difference (*p*-value < 0.05) in protein concentration between different TMP values (at a fixed pH of 5, 7, or 9) in the same row.

**Table 5:** Percentage of amino acid of hydrolysates at different pH values.

Amino Acids	Percentage Composition (%*w*/*w*)								
	pH 5			pH 7			pH 9		
	F	P	R	F	P	R	F	P	R
**Hydrophobic ** **(aliphatic)**	27.1	29.8	24.7	27.5	30.0	24.9	28.9	31.1	24.8
Valine	2.4	4.3	3.9	3.6	4.3	3.9	3.7	4.2	3.9
Isoleucine	2.2	3.8	3.6	2.8	3.8	3.6	2.9	3.7	3.5
Leucine	13.1	8.6	7.7	11.4	8.7	7.8	11.6	8.9	7.7
Methionine	3.8	3.4	2.1	3.8	3.3	1.9	4.9	3.5	2.3
Alanine	5.6	9.8	7.5	5.9	9.9	7.6	5.8	10.7	7.5
**Hydrophobic ** **(aromatic)**	1.6	8.1	6.6	5.9	7.8	6.7	5.9	8.0	6.6
Phenylalanine	0.8	4.0	3.6	3.7	4.0	3.7	3.8	4.0	3.6
Tryptophan	0.0	0.0	0.0	0.0	0.0	0.0	0.0	0.0	0.0
Tyrosine	0.8	4.1	3.0	2.3	3.8	3.0	2.1	4.1	3.0
**Polar neutral**	7.7	8.6	10.1	6.3	8.2	10.3	6.4	8.3	10.1
Threonine	4.2	4.5	5.5	3.1	4.2	5.7	3.2	4.0	5.6
Serine	2.6	4.1	3.8	2.4	4.0	3.8	2.5	4.3	3.8
Cysteine	0.9	0.0	0.8	0.8	0.0	0.8	0.7	0.0	0.7
**Electrical charged (acidic)**	22.2	17.9	24.7	15.6	18.9	24.1	14.7	16.9	24.5
Aspartic acid	13.3	8.9	11.3	6.7	8.8	11.2	6.5	8.8	11.1
Glutamic acid	9.0	8.9	13.4	8.8	10.0	12.9	8.2	8.1	13.4
**Electrical charged (basic)**	29.6	28.3	24.6	18.0	27.9	24.6	17.6	28.5	24.3
Histidine	12.3	9.6	5.3	4.2	9.7	5.4	4.0	9.6	5.3
Lysine	13.2	11.1	13.5	8.6	10.9	14.0	8.5	11.7	14.2
Arginine	4.1	7.6	5.7	5.2	7.2	5.2	5.1	7.2	4.8
**Unique**	11.8	7.4	9.3	26.7	7.3	9.6	26.6	7.1	9.7
Glycine	5.4	4.0	5.6	3.5	4.1	5.9	3.8	4.2	6.0
Proline	6.4	3.4	3.8	23.2	3.3	3.7	22.8	2.9	3.7
**Total**	100.0	100.0	100.0	100.0	100.0	100.0	100.0	100.0	100.0

F: Feed; P: Permeate; and R: Retentate.

**Table 6:** Effect of pH on the permeate-to-retentate (P/R) ratio of each amino acid type.

Amino Acids	P/R Ratio		
	pH 5	pH 7	pH 9
**Hydrophobic side chain—aliphatic**	0.37	0.40	0.38
Valine	0.34	0.36	0.33
Isoleucine	0.33	0.34	0.32
Leucine	0.34	0.38	0.35
Methionine	0.50	0.58	0.46
Alanine	0.40	0.44	0.44
**Hydrophobic side chain—aromatic**	0.38	0.39	0.37
Phenylalanine	0.34	0.36	0.34
Tryptophan	-	-	-
Tyrosine	0.43	0.42	0.42
**Polar neutral side chain**	0.26	0.27	0.25
Threonine	0.25	0.25	0.22
Serine	0.33	0.35	0.35
Cysteine	0.00	0.00	0.00
**Electrical charged side chain—acidic**	0.22	0.26	0.21
Aspartic acid	0.24	0.26	0.24
Glutamic acid	0.20	0.26	0.18
**Electrical charged side chain—basic**	0.35	0.38	0.36
Histidine	0.55	0.60	0.55
Lysine	0.25	0.26	0.25
Arginine	0.41	0.47	0.46
**Unique**	0.24	0.26	0.22
Glycine	0.22	0.23	0.21
Proline	0.28	0.29	0.24
**Total**	0.31	0.34	0.30

## Data Availability

Data are contained within the article.

## Supplementary Materials

The following supporting information can be downloaded at: https://www.mdpi.com/article/10.3390/foods12183367/s1. Figure S1. Used filter paper at different pH levels.
