# Supplementary material for: The Effect of Different pH Conditions on Peptides’ Separation from the Skipjack Dark Meat Hydrolysate Using Ceramic Ultrafiltration
Source: Foods. 2023 Sep 8;12(18):3367. doi: 10.3390/foods12183367 (PMC10528393; doi:10.3390/foods12183367)
Supplement: Supplementary file 1 [file foods-12-03367-s001.zip › foods-2572112-supplementary.pdf]

Supplementary data:

Figure S1 shows the differences in fouling thickness observed on the used filter paper at different pH values as the pH modification changed the charges of amino acids and peptides in the hydrolysate solution, leading to different charge interactions [1]. At pH 5, the used filter paper showed less fouling layer than at pH 7 and 9. This supported the increase in charged side chain amino acid compositions at feed pH 5. There were possibilities that more peptides at pH 5 passed the filter paper and remained in the pre-filtrated hydrolysate. Moreover, at this pH, high MW peptides would gain more charges and therefore exhibited less aggregation. As seen in the used filter paper, the fact that more particles were found on the filter paper at pH 7 and 9 which indicated more protein aggregation.

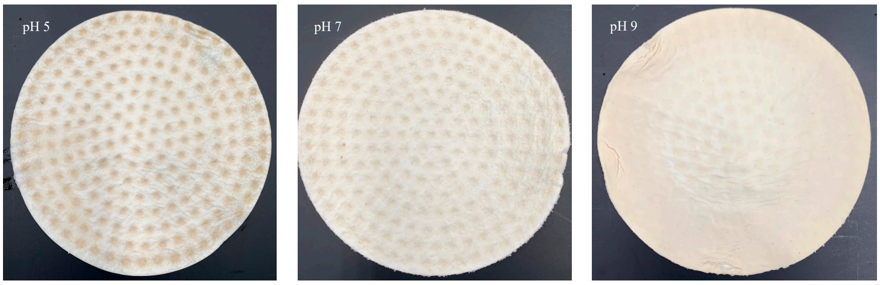

Figure S1 Used filter paper at different pH levels.

1. Groleau, P.E.; Morin, P.; Gauthier, S.F.; Pouliot, Y. Effect of physicochemical conditions on peptide-peptide interactions in a tryptic hydrolysate of beta-lactoglobulin and identification of aggregating peptides. *J. Agric. Food Chem.* **2003**, *51*, 4370–4375. https://doi.org/10.1021/jf0259720.
